# Experimental Investigation of Thrust Force, Delamination and Surface Roughness in Drilling Hybrid Structural Composites

**DOI:** 10.3390/ma14164468

**Published:** 2021-08-09

**Authors:** Vigneshwaran Shanmugam, Uthayakumar Marimuthu, Sundarakannan Rajendran, Arumugaprabu Veerasimman, Adamkhan Mahaboob Basha, Mohd Shukry Bin Abdul Majid, Rasoul Esmaeely Neisiany, Michael Försth, Gabriel Sas, Seyed Mohammad Javad Razavi, Oisik Das

**Affiliations:** 1 Department of Mechanical Engineering, Saveetha Institute of Medical and Technical Sciences, Saveetha School of Engineering, Chennai 602105, India; s.vigneshwaren@gmail.com; 2 Department of Mechanical Engineering, Kalasalingam Academy of Research and Education, Krishnankoil 626126, India; sundarakannan.r@gmail.com (S.R.); v.arumugaprabu@klu.ac.in (A.V.); adamkhanm@gmail.com (A.M.B.); 3 Faculty of Mechanical Engineering Technology, Universiti Malaysia Perlis (UniMAP), Arau 02600, Perlis, Malaysia; shukry@unimap.edu.my; 4 Department of Materials and Polymer Engineering, Faculty of Engineering, Hakim Sabzevari University, Sabzevar 9617976487, Iran; r.esmaeely@hsu.ac.ir; 5 Structural and Fire Engineering Division, Department of Civil, Environmental and Natural Resources Engineering, Luleå University of Technology, 97187 Luleå, Sweden; michael.forsth@ltu.se (M.F.); gabriel.sas@ltu.se (G.S.); 6 Department of Mechanical and Industrial Engineering, Norwegian University of Science and Technology, 7491 Trondheim, Norway

**Keywords:** hybrid structural composite, drilling, fibre, thrust force, delamination, surface roughness, design of experiments

## Abstract

Filled hybrid composites are widely used in various structural applications where machining is critical. Hence, it is essential to understand the performance of the fibre composites’ machining behaviour. As such, a new hybrid structural composite was fabricated with redmud as filler and sisal fibre as reinforcement in polyester matrix. The composite was then tested for its drilling performance. A comprehensive drilling experiment was conducted using Taguchi L27 orthogonal array. The effect of the drill tool point angle, the cutting speed, the feed rate on thrust force, delamination, and burr formation were analysed for producing quality holes. The significance of each parameter was analysed, and the experimental outcomes revealed some important findings in the context of the drilling behaviour of sisal fibre/polyester composites with redmud as a filler. Spindle speed contributed 39% in affecting the thrust force, while the feed rate had the maximum influence of ca. 38% in affecting delamination.

## 1. Introduction

Particle reinforced hybrid composites have become highly recommended materials in various structural applications due to their advanced engineering properties. These composites are gaining more attention in modern application—especially in many engineering structures and constructions—because of their light weight and increased strength over conventional materials [1,2,3]. The particle composites have increased stiffness, strength, and moisture-absorption stability, making them better candidates for structural applications [4,5,6,7]. However, machinability (e.g., drilling) of these structural composites is difficult because of their heterogeneous properties [8,9]. It is necessary to machine holes and slots in composites to create temporary joints with nuts and bolts. Loss of properties during machining/drilling reduces structural strength and lowers assembly tolerance that can be detrimental to long-term performance.

One of the main constraints that prevent these composites from widespread structural applications is the difficulty in machining [10]. In general, machining of fibre-reinforced composites produces higher delamination, which is unavoidable [11]. Reinforcement of particles in the composite may make machining more difficult, thus causing the tool to wear rapidly [12]. By controlling machining parameters, machining can be conducted with reduced delamination, surface roughness, and cutting forces [13]. The machining phenomenon of polymeric structural composites is different and more difficult than the process used for their metallic counterparts. During machining of fibre composites, the tool interacts with the fibre and matrix, which softens the matrix surface and results in fibre pullout, uncut fibres, delamination, and poor surface finish [14]. Considering the increasing structural application of these composites, the limitation in their machining performance should be envisaged in a detailed and systematic way for producing quality holes with reduced damage. Babu et al. [15] reported that delamination in fibre composites can be minimised by machining at lower feed and higher cutting speed. Chandramohan and Marimuthu [16] reported that thrust forces have no significant effect on lower feed and cutting speed. However, their investigation found that the thrust force was low at a high feed rate and cutting speed. Yallew et al. [17] reported that the tool point angle is an important factor in deciding the delamination and hole quality in natural fibre-reinforced composites and that the drilling quality of natural fibre composites can be increased through chemical treatment of fibre [18]. Athijayamani et al. [19] reported that the chemically treated fibre-reinforced composite exhibited better dimensional accuracy than the untreated fibre composite. Many researchers studied the drilling mechanism and the effect of drilling parameters on fibre-reinforced polymer composites [20,21,22,23]. Jayabal and Natarajan [21] found that the tool wear during drilling of natural fibre composites can influence the hole quality. Durão et al. [22] reported that the tool geometry and material properties have a direct impact on thrust force and delamination.

From the review, a research gap has been identified regarding particle-reinforced hybrid structural composites. In particular, the drilling effect of particle reinforcement in thrust, delamination, and surface roughness must be studied in detail. Much work has been reported on various machining characteristics of metals, alloys, and homogeneous materials; however, comparatively less has been reported on the machining performance of hybrid fibre structural composites. In fact, no particular study has been found on the polyester-based natural fibre/redmud particulate-reinforced composites. Thus, the present study attempts to fill this gap by studying the thrust, delamination, and surface roughness on the drilling of hybrid structural composites, particularly a redmud/sisal polyester composite. The addition of redmud, an industrial waste, would induce a cleaner production. Sisal fibre, one of the natural fibres, has good mechanical characteristics and is also treated with a silane chemical agent to improve the fibre matrix bonding ability. A comprehensive drilling experimental test was conducted by varying the drill tool point angle, spindle speed, and feed rate, and the significance of each parameter was analysed using Taguchi analysis. The overarching aim of the study was to comprehend the various machining parameters affecting the drilling operation of fibre-based composites used in structural applications.

## 2. Materials and Methods

Silane-treated sisal fibre were used for the fabrication of the hybrid structural composite. The composite was developed with 20 wt% redmud and 40 wt% fibre reinforcements because the author’s previous work indicated the maximum mechanical performance at this combination [24]. Sisal fibres mat was purchased from Tokyo corporation, Coimbatore, India. The purchased sisal fibres were treated with a silane coupling agent (Triethoxy(ethyl)silane, I.L.E.CO, Madurai, India). During treatment, the fibres were immersed in the silane solution prepared with triethoxy(ethyl)silane at a concentration of 2%. The immersion was continued for 2 h. After that, the fibres were taken out and sun-dried. Redmud, meanwhile, was collected from the National Aluminium Company Ltd., Damanjodi, India. The polyester resin with the catalyst (methyl ethyl before peroxide, Vasavibala Resins Private Limited, Chennai, India), along with the accelerator (cobalt naphthalate, Vasavibala Resins Private Limited, Chennai, India), were selected as the matrix material and curing agent, respectively. The polyester resin was selected because of its superior adhesion to natural fibre as well as its compatibility with filler materials. The hybrid structural composite plank/plate of size 300 mm × 127 mm × 6 mm was fabricated using the compression moulding process. To achieve the samples, a steel mould was used. A homogenous solution of redmud and polyester was prepared by mixing redmud into the polyester solution manually for 10 min. Next, the solution was applied over the fibre placed in the mould. The mould was then closed and compressed at 120 N for 5 h. After curing, the final fabricated composite was retrieved and trimmed for drilling experiments. The fabricated composite structural component properties are compared with the untreated fibre composite in Table 1.

### 2.1. Drilling Experiment

**Figure 1:**
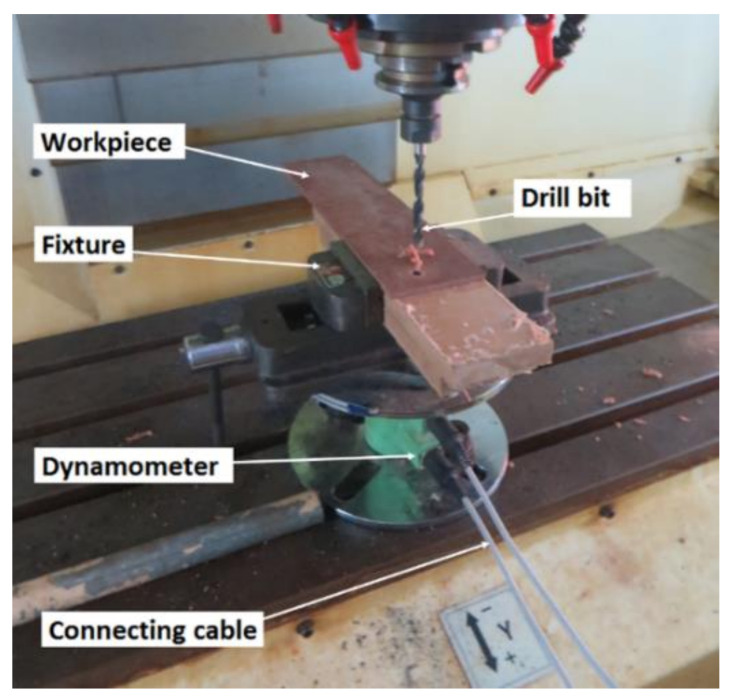
A typical structural component composite sample during the drilling process.

The drilling experiment was conducted using a numerically controlled vertical drilling machine, model JV 55, supplied by Laxmi Machine Works (Amal Jyothi College of Engineering, Kerala, India). The machine has a maximum spindle speed of 6000 rpm and cutting feed rate of 10 m/min. The experiment was conducted as per L_27_ orthogonal array by controlling spindle speed, feed rate, and tool point angle. The high-speed steel (HSS) tool of 8 mm diameter was used for machining. The thrust force developed during drilling was measured using the IEICOS drill dynamometer, model 600A, (Amal Jyothi College of Engineering, Kerala, India). The dynamometer consisted of a sensor unit and a digital force indicator with the amplifier connected to the computer system. All the experiments were conducted in a dry environment. Figure 1 shows the experimental setup during drilling.

**Figure 2:**
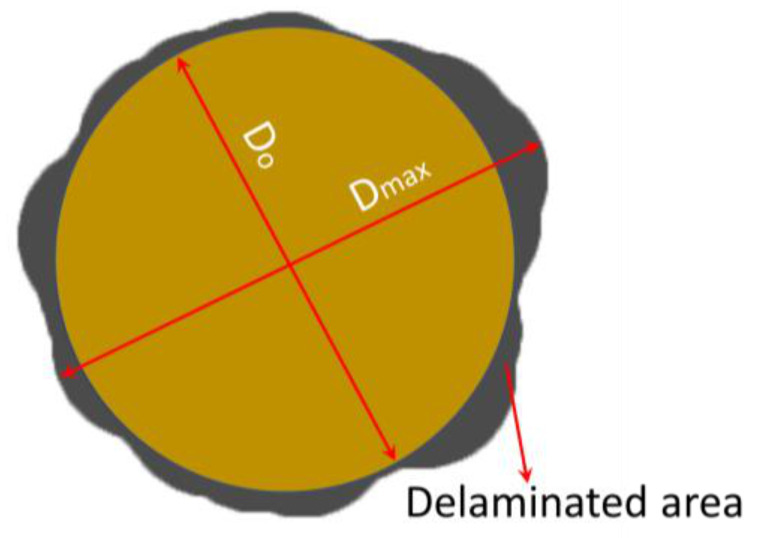
A schematic diagram of the measurement of the delamination factor.

After the drilling experiment, the drilled holes were analysed for delamination using the Motic optical microscope having in-built Moticam 2500 camera, digitally controlled by Motic Images Plus 2.0 ML image processing software (Kalasalingam Academy of Research and Education, Tamil Nadu, India). Delamination was measured at the exit side of the drilled hole by measuring the maximum delamination diameter, as shown in Figure 2.

Then the delamination factor (F_d_) was calculated using Equation (1).

(1) $$F_{d}=\frac{D_{\max}}{D_{o}}$$

where D_max_ is the diameter at maximum delamination area and D_o_ is the nominal hole diameter. Open-source software Image-J (National Institutes of Health, Bethesda, MD, USA) was used for the delamination measurement. The surface roughness of the composites was determined using the surface roughness tester Mitutoyo SJ410 (Kalasalingam Academy of Research and Education, Tamil Nadu, India) with a cut of the length of 4 mm. The surface roughness value is the average of five readings measured on the drill hole.

### 2.2. Taguchi Experimental Design

The Taguchi experimental design was selected to design and analyse the significance of the control variables on output response. Based on the number of parameters/variables, a specific orthogonal array can be chosen to identify the optimal conditions leading to a desirable outcome, as well as lower the number of required experiments. The main objectives of the Taguchi method were to save time and to produce quality outputs at low costs. Both objectives can be achieved using the orthogonal array proposed by Taguchi. The total number of experiments can be reduced by using Taguchi factorial experimental design rather than full-scale factorial analysis. Additionally, variable and detailed information regarding the influence of the parameters on the experimental results can be obtained. In the present investigation, the Standard Taguchi L27 (313) orthogonal array was selected for experimenting. Point angle, spindle speed, and feed rate were the selected control variables, and their corresponding level is shown in Table 2.

Quality characteristics of a control variable are evaluated using a signal to noise (S/N) ratio, where the signal represents the “desirable” output and noise refers to the variations in the output due to “undesirable” factors. The Taguchi method uses the signal-to-noise ratio to express the scatter around a target value. In Taguchi experimental analysis, the output response is analysed by considering three categories: “nominal is better, larger is better, and smaller is better.” In Taguchi analysis, the mean response of the experimental data was evaluated by transforming the output data to signal to noise ratio (S/N). For the present experiment, the S/N ratio of the output responses was found at a “smaller is better” condition since the rate of thrust, delamination, and Ra should be minimum. The S/N ratio for the “smaller is better” condition is expressed in the logarithmic transformation of the loss function, as in Equation (2),

(2) $$\frac{S}{N}=-10\;\mathrm{Log}\frac{1}{n}\sum y^{2}$$

where n is the number of observations, and y denotes the observed data (Equation (2)). The significance of the individual parameters was determined by the Analysis of Variance (ANOVA). The ANOVA table consists of the *p*-value, F-value, and percentage (%) of the contribution of each parameter. The larger the F-value of a parameter, the more notable it is. The significance of a parameter can also be understood by its probability value. If the *p*-value is less than 0.05 for a parameter, then it is significant in the process.

## 3. Results and Discussion

For the experimentation results, the thrust force, delamination factor, and surface roughness were analysed and reported in the Table 3.

### 3.1. Thrust Force Analysis

**Figure 3:**
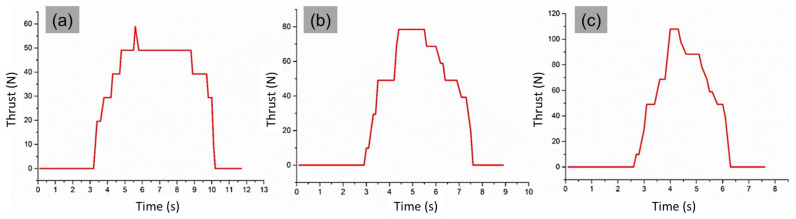
Drilling cycles for specimens machined with 90° point angle and spindle speed of 2000 rpm with a (**a**) feed rate 100 mm/min and thrust force 58.56 N, (**b**) feed rate 150 mm/min and thrust force 78.48 N, and (**c**) feed rate 200 mm/min and thrust force 107.91 N.

The thrust force variation with time is shown in Figure 3. At the beginning of the drilling cycle, the cutting lips were active in material removal; therefore, the thrust force quickly reached its peak. The cutting lips and chisel edge of the tool directly affected the thrust since it was more important in material removal. The chisel edge of the tool did not cut the material but removed the formed chip material by extrusion action. Once the through-hole is done, the lip and chisel edges of the tool were free from machining, and the thrust became zero.

**Figure 4:**
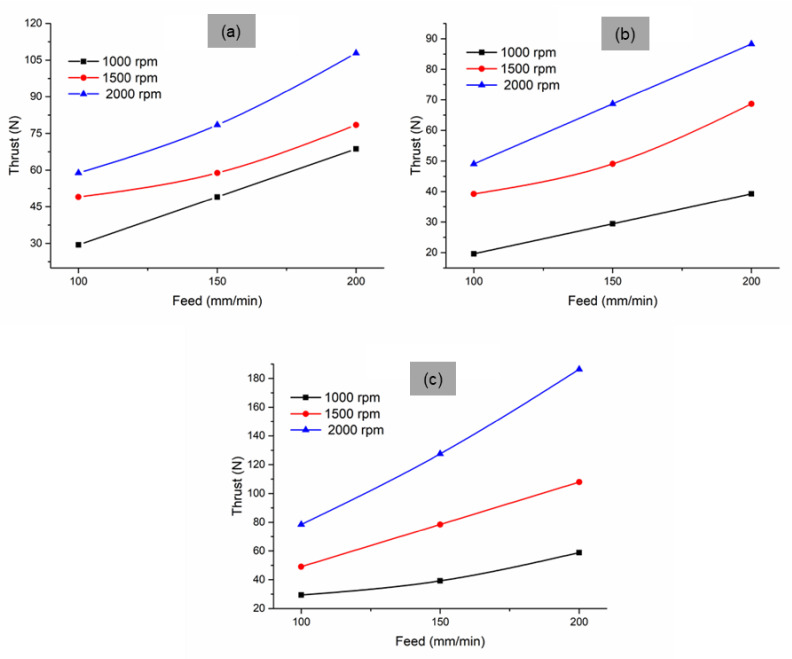
Variation in thrust force on redmud-filled sisal composite structures at different point angles: (**a**) 90°, (**b**) 118°, and (**c**) 135°.

The effect of drilling variables on the thrust force is shown in Figure 4. The generated thrust increased along with the increase in the feed rate and spindle speed. This was also evident from the ANOVA results in Table 4. The ANOVA analysis shows the significance of the three parameters affecting the thrust force. F-values of point angle, spindle speed, and feed were 27.43, 68.18, and 47.81, respectively, and their corresponding *p*-values for all the parameters were 0. F-value and *p*-value show that all three parameters influence the thrust force variation, with the spindle speed influencing it the most. The spindle speed contributed 39.23% in affecting the thrust, followed by the feed rate at 27.51% and the point angle at 15.78%. On varying the point angle, the thrust force was higher at 135° point angle, followed by 90° point angle. The drill tool with a 118° point angle generated a lower thrust force. During drilling with 90° and 135° point angles, a higher strain was developed in the composite, which was responsible for the increased thrust. At 118° point angle, the composite exhibited lower axial pressure, which reduced the generated thrust force. The variation in the thrust force due to the change in the spindle speed and feed rate were quite similar in the three different point angle tools. The increase in the feed rate increased the thrust force. This was due to higher resistance to the chip formation. At a high feed rate, the material was not fully removed, and chip formation was affected. Therefore, the resistance force developed against the tool, resulting in increased thrust force. At all the point angles, an increase in the spindle speed increased the thrust. This was because the presence of redmud particles in the matrix acted as a barrier to the progress of the drill tool and increased the thrust even at high speed. This also may have been due to an increase in heat generation during drilling. The heat generated may have been concentrated around the drill area, which made the matrix, together with the redmud particles, stick to the tool, thus developing the buildup edge. The developed buildup edge also may be a reason for the increased thrust at high speed.

### 3.2. Delamination Analysis

Table 5 shows the calculated ANOVA results of the delamination factor. The ANOVA results show that the delamination is mostly affected by the point angle. The ANOVA analysis shows the significance of the three parameters in affecting delamination. F-values of point angle, spindle speed, and feed were 383.89, 76.62, and 118.07, respectively, and their corresponding *p*-values were 0.001, 0.194, and 0. F-value and *p*-value show that the point angle contributed more to affecting the delamination factor. Thus, the 63.05% contribution of the point angle is the primary factor in deciding the delamination factor, followed by the feed rate of 19.39% and spindle speed of 12.58%.

**Figure 5:**
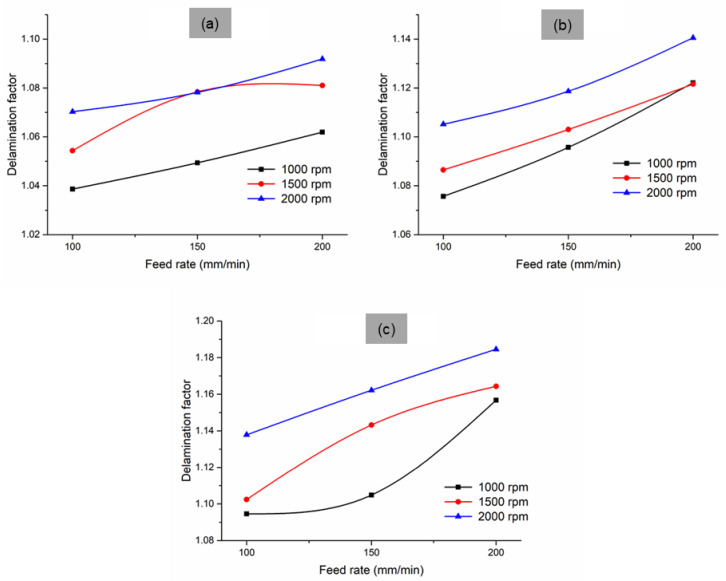
Variation in delamination factor on redmud-filled sisal composite structures at different point angles: (**a**) 90°, (**b**) 118°, and (**c**) 135°.

Figure 5 shows that the increase in the feed rate also increased the delamination, while an increase in the spindle speed also increased the delamination irrespective of point angle. This characteristic is due to the variation in the cutting temperature. In other words, the cutting temperature produced at lower spindle speed is minimum, and the delamination due to this condition formed because of the shearing stress-induced while drilling. At a high feed rate, the composite surface exhibited high stress due to interference from the tool. Therefore, when the tool started penetrating the composite surface, the material surface near the tool edge developed higher stress against penetration. This created high delamination at the entry side of the tool. At a high feed rate, the tool also wore due to the formation of buildup edges [21], which directly corresponded to the increase in delamination. However, at high speeds, the developed buildup edges were removed due to the generation of high temperatures. In addition, at high speeds, the matrix softened due to the increased temperature, which contributed to the rapid cutting of surface and chip removal without any delamination. It can be justified that the lower feed rate and higher spindle speed were suitable for producing holes with reduced delamination. According to the work of Athijayamani et al. [19], the hole quality is the function of matrix and fibre properties. Therefore, delamination can be reduced in the fibre composites by controlling the fibre and matrix ratio, as well as by improving the interfacial bonding.

### 3.3. Surface Roughness (R_a_) Analysis

**Figure 6:**
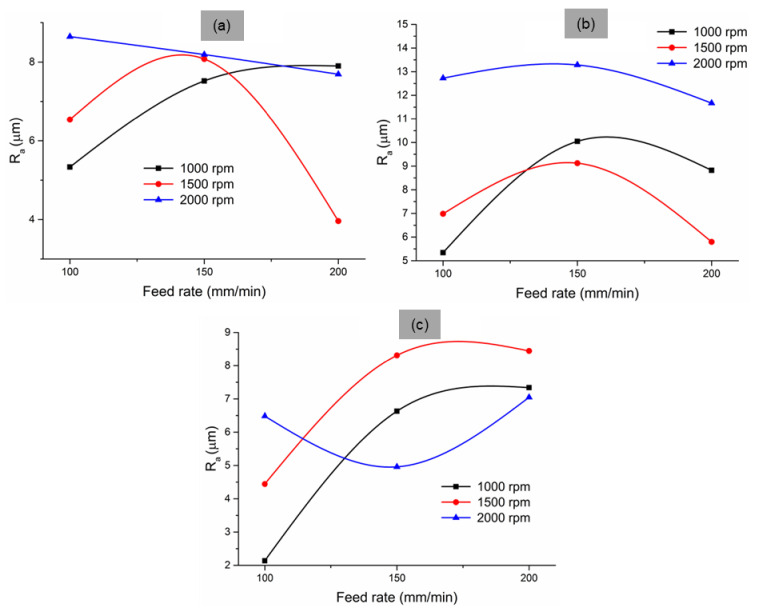
The variation in surface roughness on redmud-filled sisal composite structures at different point angles: (**a**) 90°, (**b**) 118°, and (**c**) 135°.

In the present investigation, surface roughness found on the drilled surface is described by R_a_ (the average surface roughness). Surface roughness variation with respect to the point angle, spindle speed, and feed rate is shown in Figure 6.

**Figure 7:**
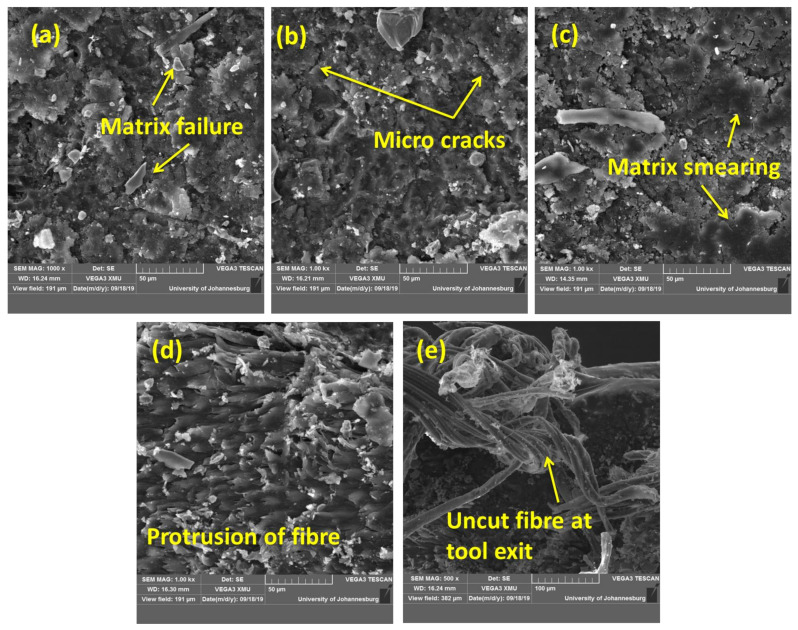
The morphology of a drilled hole in the composite structures. (**a**) Matrix failure, (**b**) Micro cracks, (**c**) Matrix smearing, (**d**) Fibre protrusion, (**e**) Uncut fibres.

Figure 6 shows that the roughness values were scattered; as such, the surface roughness values did not follow the trend on variation with drilling parameters. This is because of the non-homogeneous property of the composite. The presence of fibre and particles in the composite surface was not uniform. Under drilling, the surface may become more irregular due to the different cut mechanisms involved (Figure 7). The matrix material failed under high stress, while the fragmented matrix was found stuck to the cut surface (Figure 7a). The matrix cracks were noted on the surface due to the high stress developed in the drill face during tool penetration (Figure 7b). Due to high heat generation, the matrix became molten and smeared on the wall of the cut surface (Figure 7c). The high drill force caused the fibres to pull out, protrude from and become deboned from the matrix (Figure 7d). In some regions, the fibres were broken and fragmented, while uncut fibres (Figure 7e) were also noticed at the exit point. These variations in the failure mechanism are the reason for the irregularity and scattering in the surface roughness value. Therefore, it can be concluded that, although it is preferable in metal matrix composites, the surface roughness cannot be considered a factor in defining the drilling hole quality in these types of composite structures. Similar findings were reported by Durão et al. [25].

Table 6 shows ANOVA analysis and the significance of the three parameters in affecting surface roughness. F-value of point angle, spindle speed, and feed were 31.18, 18.69, and 11.59, respectively, and their corresponding *p*-values were 0, 0.001 and 0.004, which show that the point angle is statistically more significant for surface roughness. Point angle contributed 27.91% in affecting the surface roughness, followed by the spindle speed (16.73%) and feed (10.38%).

**Figure 8:**
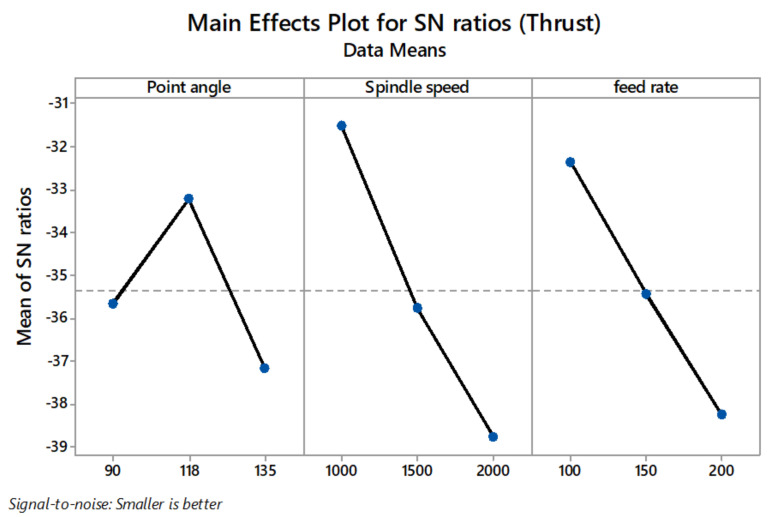
Main effect plot for S/N ratios of thrust force.

**Figure 9:**
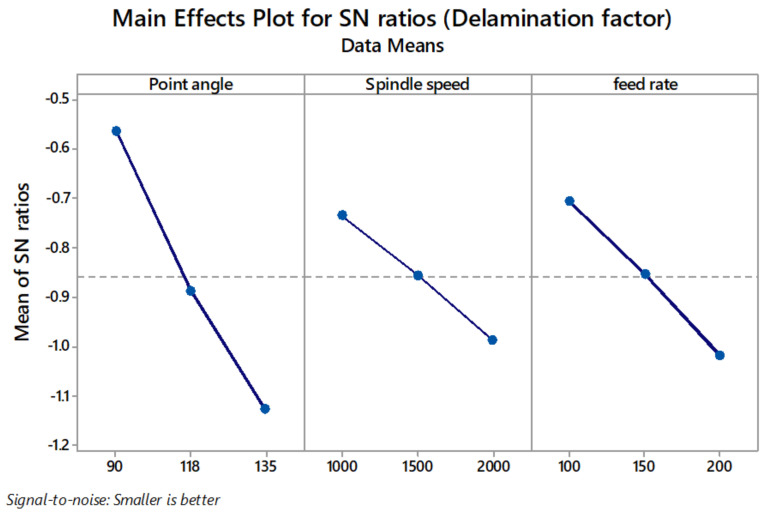
Main effect plot for S/N ratios of delamination factor.

**Figure 10:**
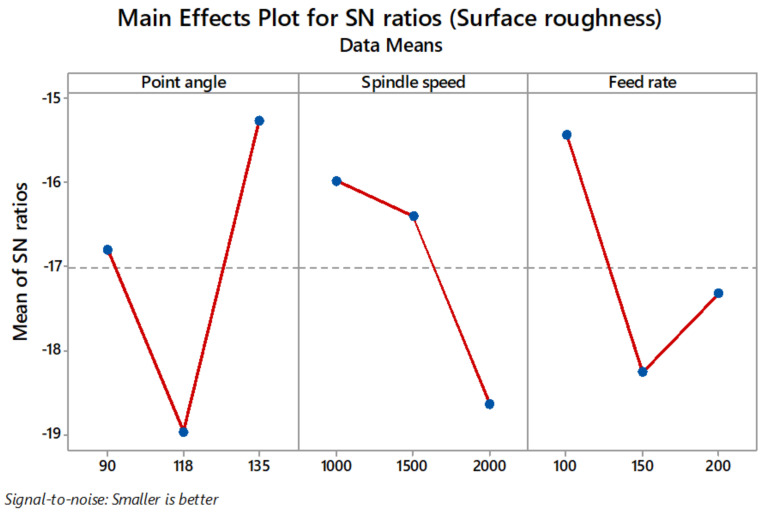
Main effect plot for S/N ratios of surface roughness (R_a_).

Table 7 shows the S/N ratio response table. Delta values in the response table help in identifying the drilling parameters. Based on the delta values, ranks have been given to variables in the order of their significance in the minimisation of thrust force, delamination factor, and surface roughness. In this regard, the minimum thrust force value was obtained at point angle 118°, spindle speed 1000 rpm, and feed rate 100 mm/min (Figure 8). The minimum delamination factor value was obtained at point angle 90°, spindle speed 1000 rpm, and feed rate 100 mm/min (Figure 9). The minimum R_a_ value was obtained at point angle 135°, spindle speed 1000 rpm, and feed rate 100 mm/min (Figure 10).

## 4. Conclusions

This study was conducted to analyse the drilling performance of silane-treated sisal/redmud hybrid structural composite on varying point angles, spindle speeds, and feed rates. Thrust force, exit delamination, and surface roughness were investigated using Taguchi analysis. Based on the results, the following conclusions were drawn:

- The thrust force increased with the increase in the feed rate and spindle speed. Compared to spindle speed, thrust force was highly influenced by feed rate. Drill tool with 118° point angle generated lower thrust force. ANOVA results showed that the spindle speed (39.23%) produced the maximum contribution affecting the thrust, followed by the feed rate (27.51%) and point angle (15.78%).
- Delamination was highly influenced by the feed rate (37.99%). Point angle 90° showed maximum delamination, while minimum delamination was noted at 118°. Variation in spindle showed minor variation in delamination.
- A quality hole with minimum thrust force was obtained at point angle 118°, spindle speed 1000 rpm, and feed rate 100 mm/min.
- The minimum delamination factor value was obtained at point angle 90°, spindle speed 1000 rpm, and feed rate 100 mm/min.
- The minimum surface roughness value was obtained at point angle 135°, spindle speed 1000 rpm, and feed rate 100 mm/min.

According to the findings, redmud-based composites have improved machinability characteristics, which may increase the likelihood of these composites being used in structural applications. This research could pave the way for a better understanding of the machinability behaviour of particle based structural composites.

## Figures and Tables

**Table 1:** Properties of the fabricated composite structure [23].

Property	Untreated Sisal Fibre/Redmud Composite	Silane Treated Sisal Fibre/Redmud Composite
Tensile strength (MPa)	49	63
Flexural strength (MPa)	199	244
Impact strength (J/m)	20	26
Hardness (shore D)	78	90
Density (g/cm^3^)	1.4	1.5
Voids (%)	3.5	1.2

**Table 2:** Process variables and their levels.

Symbol	Process Variables	Levels		
		I	II	III
A	Point angle (°)	90	118	135
B	Spindle Speed (rpm)	1000	1500	2000
C	Feed rate (mm/min)	100	150	200

**Table 3:** Experiment results.

Ex. No	Point Angle (°)	Spindle Speed (rpm)	Feed Rate (mm/min)	Thrust (N)	S/N Ratio of Thrust	F_d_	S/N Ratio of F_d_	R_a_ (µm)	S/N Ratio of R_a_
1	90	1000	100	29.43	−29.38	1.039	−0.329	5.332	−14.538
2	90	1000	150	49.05	−33.81	1.049	−0.419	7.521	−17.526
3	90	1000	200	68.67	−36.74	1.062	−0.522	7.900	−17.953
4	90	1500	100	49.05	−33.81	1.054	−0.459	6.538	−16.308
5	90	1500	150	58.86	−35.40	1.078	−0.655	8.078	−18.146
6	90	1500	200	78.48	−37.90	1.081	−0.677	3.961	−11.956
7	90	2000	100	58.86	−35.40	1.070	−0.590	8.646	−18.736
8	90	2000	150	78.48	−37.90	1.078	−0.653	8.191	−18.267
9	90	2000	200	107.91	−40.66	1.092	−0.764	7.692	−17.721
10	118	1000	100	19.62	−25.85	1.076	−0.634	5.341	−14.553
11	118	1000	150	29.43	−29.38	1.096	−0.794	10.048	−20.042
12	118	1000	200	39.24	−31.87	1.122	−1.001	8.824	−18.913
13	118	1500	100	39.24	−31.87	1.086	−0.721	6.982	−16.880
14	118	1500	150	49.05	−33.81	1.103	−0.851	9.124	−19.204
15	118	1500	200	68.67	−36.74	1.122	−0.997	5.798	−15.265
16	118	2000	100	49.05	−33.81	1.105	−0.868	12.726	−22.094
17	118	2000	150	68.67	−36.74	1.119	−0.974	13.286	−22.468
18	118	2000	200	88.29	−38.92	1.141	−1.142	11.672	−21.343
19	135	1000	100	29.43	−29.38	1.095	−0.785	2.136	−6.590
20	135	1000	150	39.24	−31.87	1.105	−0.866	6.630	−16.430
21	135	1000	200	58.86	−35.40	1.157	−1.265	7.339	−17.312
22	135	1500	100	49.05	−33.81	1.102	−0.847	4.442	−12.952
23	135	1500	150	78.48	−37.90	1.143	−1.163	8.313	−18.395
24	135	1500	200	107.91	−40.66	1.164	−1.321	8.447	−18.534
25	135	2000	100	78.48	−37.90	1.138	−1.122	6.484	−16.237
26	135	2000	150	127.53	−42.11	1.162	−1.305	4.958	−13.906
27	135	2000	200	186.39	−45.41	1.185	−1.471	7.047	−16.960

F_d_—Delamination factor, R_a_—Surface roughness, S/N—Signal to noise ratio.

**Table 4:** ANOVA results of thrust force analysis.

Source	DF	Seq SS	Contribution	Adj SS	Adj MS	F-Value	*p*-Value
Point angle	2	5182.5	15.78%	5182.5	2591.25	27.43	0.00
Spindle speed	2	12,881.4	39.23%	12,881.4	6440.69	68.19	0.00
Feed rate	2	9031.9	27.51%	9031.9	4515.97	47.81	0.00
Point angle × Spindle speed	4	2901.3	8.84%	2901.3	725.34	7.68	0.008
Point angle × Feed rate	4	1040.8	3.17%	1040.8	260.19	2.75	0.104
Spindle speed × Feed rate	4	1040.8	3.17%	1040.8	260.19	2.75	0.104
Error	8	755.6	2.30%	755.6	94.45	-	-
Total	26	32,834.3	100.00%	-	-	-	-

Adj—Adjusted, DF—Degree of Freedom, SS—Sum of squares, MS—Mean square.

**Table 5:** ANOVA results of delamination factor analysis.

Source	DF	Seq SS	Contribution	Adj SS	Adj MS	F-Value	*p*-Value
Point angle	2	0.023368	63.05%	0.023368	0.011684	383.89	0.00
Spindle speed	2	0.004664	12.58%	0.004664	0.002332	76.62	0.00
Feed rate	2	0.007187	19.39%	0.007187	0.003594	118.07	0.00
Point angle × pindle speed	4	0.000451	1.22%	0.000451	0.000113	3.7	0.054
Point angle × Feed	4	0.00086	2.32%	0.00086	0.000215	7.06	0.01
Spindle speed × Feed	4	0.000291	0.78%	0.000291	0.000073	2.39	0.137
Error	8	0.000243	0.66%	0.000243	0.00003	-	-
Total	26	0.037065	100.00%	-	-	-	-

Adj—Adjusted, DF—Degree of Freedom, SS—Sum of squares, MS—Mean square.

**Table 6:** ANOVA results of surface roughness analysis.

Source	DF	Seq SS	Contribution	Adj SS	Adj MS	F-Value	*p*-Value
Point angle	2	46.191	27.91%	46.191	23.0957	31.18	0
Spindle speed	2	27.685	16.73%	27.685	13.8424	18.69	0.001
Feed rate	2	17.179	10.38%	17.179	8.5893	11.59	0.004
Point angle × Spindle speed	4	31.124	18.80%	31.124	7.781	10.5	0.003
Point angle × Feed rate	4	13.36	8.07%	13.36	3.3401	4.51	0.034
Spindle speed × Feed rate	4	24.048	14.53%	24.048	6.0119	8.12	0.006
Error	8	5.926	3.58%	5.926	0.7408	-	-
Total	26	165.513	100.00%	-	-	-	-

Adj—Adjusted, DF—Degree of Freedom, SS—Sum of squares, MS—Mean square.

**Table 7:** S/N ratio response table.

Level	Thrust			Delamination Factor			Surface Roughness (R_a_)		
	Point Angle	Spindle Speed	Feed	Point Angle	Spindle Speed	Feed	Point Angle	Spindle Speed	Feed
1	−32.66	−28.51	−29.35	32.69	20.71	22.89	−16.79	−15.98	−15.43
2	−30.21	−32.76	−32.43	25.62	32.71	32.71	−18.97	−16.4	−18.26
3	−34.15	−35.75	−35.24	42.53	47.43	45.25	−15.26	−18.64	−17.33
Delta	3.94	7.24	5.89	16.91	26.72	22.36	3.72	2.65	2.83
Rank	3	1	2	3	1	2	1	3	2

## Data Availability

All the data is available within the manuscript.
